# Using Sparfloxacin-Capped Gold Nanoparticles to Modify a Screen-Printed Carbon Electrode Sensor for Ethanol Determination

**DOI:** 10.3390/s23198201

**Published:** 2023-09-30

**Authors:** Vasanth Magesh, Vishaka S. Kothari, Dhanraj Ganapathy, Raji Atchudan, Sandeep Arya, Deepak Nallaswamy, Ashok K. Sundramoorthy

**Affiliations:** 1 Centre for Nano-Biosensors, Department of Prosthodontics, Saveetha Institute of Medical and Technical Sciences, Saveetha Dental College and Hospitals, 162 Poonamallee High Road, Velappanchavadi, Chennai 600077, India; 2 School of Chemical Engineering, Yeungnam University, Gyeongsan 38541, Republic of Korea; 3 Department of Physics, University of Jammu, Jammu 180006, India

**Keywords:** gold nanoparticles, sparfloxacin, chemical synthesis, screen-printed electrode, ethanol sensor

## Abstract

Alcohol is a dangerous substance causing global mortality and health issues, including mental health problems. Regular alcohol consumption can lead to depression, anxiety, cognitive decline, and increased risk of alcohol-related disorders. Thus, monitoring ethanol levels in biological samples could contribute to maintaining good health. Herein, we developed an electrochemical sensor for the determination of ethanol in human salivary samples. Initially, the tetra-chloroauric acid (HAuCl_4_) was chemically reduced using sparfloxacin (Sp) which also served as a stabilizing agent for the gold nanoparticles (AuNPs). As-prepared Sp-AuNPs were comprehensively characterized and confirmed by UV-visible spectroscopy, X-ray diffraction, field emission scanning electron microscopy (FE-SEM), energy-dispersive X-ray spectroscopy (EDS), and elemental mapping analysis. The average particle size (~25 nm) and surface charge (negative) of Sp-AuNPs were determined by using dynamic light scattering (DLS) and Zeta potential measurements. An activated screen-printed carbon electrode (A-SPE) was modified using Sp-AuNPs dispersion, which exhibited greater electrocatalytic activity and sensitivity for ethanol (EtOH) oxidation in 0.1 M sodium hydroxide (NaOH) as studied by cyclic voltammetry (CV) and differential pulse voltammetry (DPV). DPV showed a linear response for EtOH from 25 µM to 350 µM with the lowest limit of detection (LOD) of 0.55 µM. Reproducibility and repeatability studies revealed that the Sp-AuNPs/A-SPEs were highly stable and very sensitive to EtOH detection. Additionally, the successful electrochemical determination of EtOH in a saliva sample was carried out. The recovery rate of EtOH spiked in the saliva sample was found to be 99.6%. Thus, the incorporation of Sp-AuNPs within sensors could provide new possibilities in the development of ethanol sensors with an improved level of precision and accuracy.

## 1. Introduction

Nanomaterials have unique features due to their small size, higher surface-area-to-volume-ratio, surface tunability, and distinct electrical behavior, which make them useful and allow them to offer various prospects for diverse and cutting-edge applications [1,2]. In particular, gold nanoparticles (AuNPs) are very attractive for nanotechnology research due to their enticing properties of size-dependent color change [3], high surface area [4], high stability [5], unique optical properties [3,6], low toxicity, excellent biocompatibility [7], high thermal conductivity [8], and efficient catalytic activity [9,10]. They have a broad spectrum of applications, including sensors, energy storage, bio-imaging, drug delivery, and optoelectronics [11,12,13].

Nowadays, multiple techniques are available for producing AuNPs, including chemical synthesis (using an inorganic or organic compound as a reducing as well as stabilizing agent) [14,15], electrochemical synthesis (using an electrochemical method to reduce the gold ions onto the electrode surface) [16,17], green synthesis (using a plant leaf, fruit, and flower extract as a reducing agent) [18,19,20], photochemical synthesis (using light energy to reduce gold ions) [21,22], and bio-inspired synthesis (using bacteria or enzymes to produce AuNPs) [23,24,25,26]. Among these, the chemical synthesis process entails using a reducing agent to reduce an aqueous gold precursor, which might be gold chloride or gold nitrate. For examples, sodium borohydride (NaBH_4_) [27], ascorbic acid [28], sodium citrate [29], citric acid [30], and sodium dodecyl sulphate (SDS) [31] have been used as reducing agents. The chemical reduction of the gold precursor results in the formation of AuNPs with sizes ranging from 1 to 100 nm, depending on the experimental circumstances such as the reducing agent, pH, temperature, and reactant concentration [32,33]. Citric acid was employed by Turkevich et al. in 1951 to reduce hydrogen tetrachloroaurate (HAuCl_4_) in boiling water to produce AuNPs [34]. Further, Frens improved this method by adjusting the gold-to-citrate ratio to control particle size [35]. Although larger AuNPs (such as those with a diameter of 100 nm) can be prepared, this method was most typically employed to produce diluted solutions of moderately stable spherical AuNPs with sizes ranging from 10 to 20 nm. Brust and Schriffin made significant advancements in the production of AuNPs in 1994 by adopting a biphasic reduction technique with NaBH_4_ and phase transfer reagent (tetraoctylammonium bromide) to create alkanethiol-stabilized AuNPs [36]. This method produced low-solubility AuNPs with sizes ranging from 1.5 to 5 nm by varying the reaction conditions of gold-to-thiol ratio, temperature, and rate of reaction.

All of the breakthroughs outlined above still have difficulty in producing stable AuNPs. In some cases, the stabilizing agent employed to stabilize the nanoparticles may not be suitable for the particle’s designated application [37,38]. Furthermore, controlling particle size is highly important [39]. Nanoparticles smaller than 10 nm are reported to have an enhanced electrochemical performance as well as greater sensitivity and precision in labeling techniques [40]. Although tiny particles are less stable and more prone to aggregation [41,42], most of the techniques for producing smaller AuNPs described above resulted in unstable colloids, which aggregate quickly and are not advantageous for a variety of applications. Furthermore, several AuNP stabilization procedures have been reported to prepare stable colloidal solutions [43,44], but they are inappropriate for surface modification and sensing applications because of the ionic repulsion and denaturing effects of SDS like proteins and enzymes [45,46].

On the other hand, ethanol is a hazardous substance, and the regular use of alcohol can lead to conditions like depression, anxiety, and cognitive decline, increasing the risk of alcohol-related disorders. Besides, auto-brewery syndrome (ABS), also called as gut fermentation syndrome, involves the production of ethanol within the body due to fermentation by fungi or bacteria in the digestive system [47]. While small amounts of ethanol are normally produced during digestion, when these fermenting microorganisms become pathogenic, it can lead to extremely high blood alcohol levels. This condition is more common in individuals with certain health conditions like diabetes, obesity, and Crohn’s disease, but can also affect otherwise healthy people [48,49,50]. Moreover, in postmortem cases, ethanol is the most usually encountered toxicological substance. Ethanol determination is vital, as ethanol might be a potential cause or contributing factor to the individual’s death [51,52,53]. So, the selective determination of ethanol in biological samples (breath, saliva, blood, and/or urine) can be useful for various investigations and a healthy lifestyle [54].

Sparfloxacin is an anti-bacterial drug with broad activity against common infections as well as Gram-positive and Gram-negative bacteria. It contains quinolones which act as a reducing and stabilizing agent in the synthesis of nanoparticles [55,56]. Herein, we employed sparfloxacin as a reducing agent to convert gold ions into AuNPs. The synthesized AuNPs were more stable in distilled water (DI water) and had superior electrocatalytic activity towards ethanol oxidation. When compared to alternative synthesis methods, this new process is simple and fast, and able to produce AuNPs with greater stability at low cost [57,58]. Moreover, the synthesized AuNPs through sparfloxacin (Sp-AuNPs) is a promising method which can be used for the development of highly sensitive SPE-based electrochemical sensor for ethanol determination in human salivary samples.

## 2. Experimental

### 2.1. Chemicals and Materials

Gold chloride trihydrate (tetrachloroauric acid) (HAuCl_4_, purity of ~49%), magnesium chloride (MgCl_2_, purity of 98%), and ascorbic acid (purity of 99.7%) were purchased from SRL (Sisco Research Laboratories) Pvt. Ltd., Mumbai, India. Sparfloxacin (purity of 98%), sodium hydroxide (NaOH, purity of 97%), and hydrochloric acid (HCl, purity of 35%) were obtained from Sigma-Aldrich, India. Calcium chloride (fused) LR (CaCl_2_, purity of 98%) was obtained from SD Fine-Chem Ltd., Mumbai, India. Dextrose (glucose) (purity of 99.5%) and sodium chloride (NaCl, purity of 98%) were obtained from Merck, India. All the chemicals and reagents were utilized without any additional purification. Double distilled water (Milli-Q water) was used to prepare gold solutions and electrolytes. Gold chloride solutions were stored in the dark. Distilled water (DI water, 18.2 MΩ cm) was acquired from the Millipore ultrapure water system. Other solutions and buffers were prepared according to the standard laboratory protocols.

### 2.2. Characterizations

The structure and surface morphology of AuNPs were investigated using a field emission scanning electron microscope (FE-SEM, JSM IT800 (JEOL, Tokyo, Japan)). The data for the elemental mapping (E-Map) and energy dispersive X-ray spectrum (EDS) were obtained using XPLORE-30 (Oxford, High Wycombe, UK). The particle size and surface charge of the AuNPs were investigated using DLS (dynamic light scattering) spectroscopy and a Zeta potential analyzer (Horiba nano-Partica SZ-100, Kyoto, Japan). The absorption spectrum of the AuNPs was examined using UV-visible spectroscopy (UV-Vis, Jasco, Tokyo, Japan). D8-Advance XRD equipment (BRUKER, Billerica, MA, USA) was used for collecting X-ray diffraction (XRD) data. All the electrochemical measurements, such as cyclic voltammetry (CV), electrochemical impedance spectroscopy (EIS), and differential pulse voltammetry (DPV), were carried out utilizing a CHI-760E electrochemical workstation (CH Instruments, Austin, TX, USA) with a three-electrode setup of reference electrode (Ag/AgCl soaked in 3 M KCl), counter electrode (Pt wire), and working electrode (SPE with working area of 0.07 cm^2^).

### 2.3. Preparation of Gold Nanoparticles (Sp-AuNPs)

**Figure 1:**
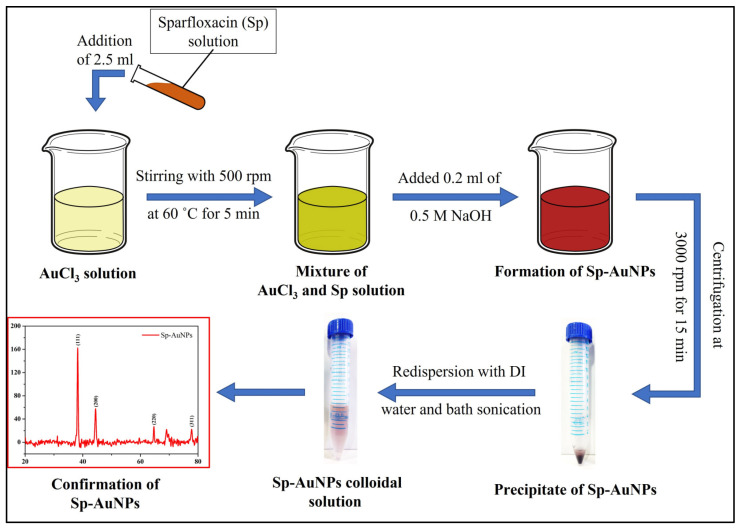
Schematic illustration of chemical synthesis of Sp-AuNPs.

The Sp-AuNPs were synthesized using a chemical reduction method [59] (as illustrated in Figure 1). In brief, the 5 mg gold chloride trihydrate (HAuCl_4_.3H_2_O) was dispersed in 5 mL DI water (1 mg/mL) and mixed well using magnetic stirrer at 500 rpm for 5 min at 60 °C (transparent yellow color solution observed). After that, 2.5 mL of sparfloxacin (Sp) solution (10 mL distilled water + 0.1 M HCL (30 µL) + 2 mM sparfloxacin (7.85 mg)) was added dropwise into the gold chloride solution with continuous stirring at 500 rpm at 60 °C for 5 min. After the addition of Sp, the transparent yellow color solution changed into a glittery goldish yellow. Finally, 0.2 mL of 0.5 M NaOH was added drop by drop with constant stirring at 500 rpm at 60 °C. After the addition of a few drops of NaOH, the goldish yellow color solution changed into a red wine color, which confirmed the formation of Sp-AuNPs. Then, the Sp-AuNP solution was cooled down to RT. After that, the solution was centrifuged for 10 min at 3000 rpm to separate the precipitate (red) and the supernatant (yellow) solution. The Sp-AuNP precipitate was then redispersed in 5 mL of DI water and sonicated for 5 min at RT. Following that, the redispersed Sp-AuNPs appeared as a transparent reddish pink color solution. The appearance of reddish pink color indicated the formation of stable colloidal Sp-AuNPs, which were then characterized and utilized to develop the ethanol sensor.

### 2.4. Fabrication of Ethanol Sensor

Firstly, the screen-printed carbon electrode (SPE) was carefully cleaned using ethanol by water bath sonication for 5 min and rinsed with DI water [60,61]. Following that, the SPE was activated (or anodized) by cyclic voltammetry (CV) in 0.1 M H_2_SO_4_ for 20 consecutive potential cycles at a scan rate of 50 mV s^−1^ in the potential window of −1.0 to +1.0 V.

**Figure 2:**
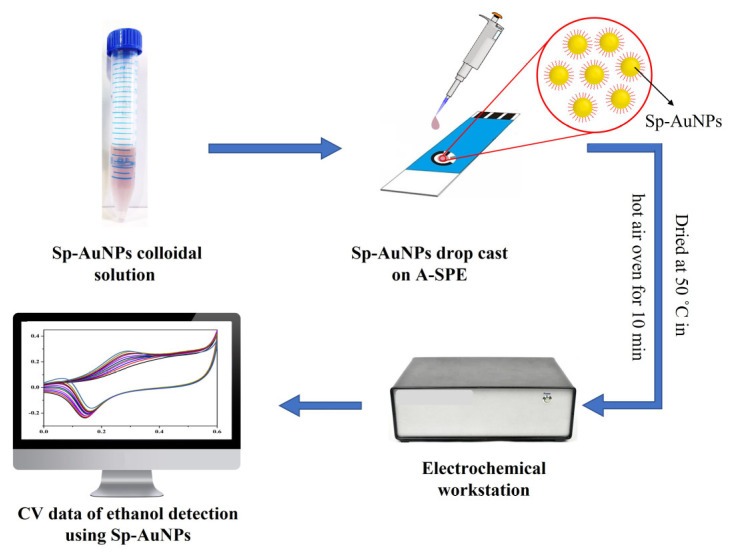
Schematic illustration of Sp-AuNPs/A-SPE sensor fabrication and ethanol determination.

The activated SPE (A-SPE) surface was then drop-casted with 7 µL of the Sp-AuNPs colloidal solution and dried in an oven with hot air at 50 °C (Figure 2). Then, the weakly bounded particles on the surface of the Sp-AuNPs/A-SPE were gently removed by dipping in DI water. Finally, the so-prepared Sp-AuNPs/A-SPE was used for ethanol (EtOH) sensing.

### 2.5. Electrochemical Measurements

CV and electrochemical impedance spectroscopy (EIS) experiments were performed using both an activated and bare SPE in 0.1 M KCl with 2 mM [Fe(CN)_6_]^3−^ in the potential window of −0.1 to +0.6 V. Compared to the bare SPE, the activated SPE (A-SPE) exhibited a higher redox peak current (I_pa_ and I_pc_), and also a lower anodic-to-cathodic peak separation for the [Fe(CN)_6_]^3−^ redox system. This observation indicated that the anodization process resulted in the generation of more electroactive sites on the SPE’s surface (Supplementary Materials: Figure S1) [62]. Furthermore, these results were congruent with the EIS data. The Nyquist plots showed a semicircle and a straight line, which corresponded to charge transfer resistance (R_ct_) and a diffusion-controlled redox process, respectively [63]. A higher R_ct_ value indicated poor electron transfer in the system. Based on the R_ct_ values, the A-SPE was found to be the best electrode with a higher electron transfer rate compared to the bare SPE (Supplementary Materials: Figure S2). It was due to the formation of electroactive edge plane sites on the A-SPE’s surface which facilitated the lateral charge transfer and enhanced overall electrochemical activity [62,64]. Next, the electrochemical behavior of EtOH on the modified electrode surface was evaluated using CV in 0.1 M NaOH in the potential window of 0 V to +0.6 V at a scan rate of 50 mV s^−1^. Under identical conditions, the CV results of the A-SPE and Sp-AuNPs/A-SPE were recorded in the same electrolyte with different concentrations of EtOH. The linear range of EtOH (25 µM–350 µM) determination was performed by using the DPV technique. DPV parameters, including pulse amplitude, potential step amplitude, pulse width, and pulse period, were set as 50 mV, 4 mV, 60 ms, and 0.5 s, respectively. Oxidation peak currents (I_pa_) vs. EtOH concentration were used to draw a calibration plot (three measurements of the DPV were made (n = 3), and the mean value as well as the standard deviation were displayed). The stability, repeatability, and reproducibility of the Sp-AuNPs/A-SPE for EtOH sensing was further investigated using CV in 0.1 M NaOH.

### 2.6. Preparation of Real-World Sample

A salivary sample was collected from an individual (aged 25 years old) who had not consumed alcohol products. The collected saliva was initially diluted with DI water at a 1:1 ratio (1 mL saliva and 1 mL DI water) and centrifuged at 3000 rpm for 5 min to remove debris and macro-molecules. Following the centrifugation process, the supernatant was collected and further diluted at 1:9 ratio (0.1 mL saliva and 0.9 mL DI water). Subsequently, the prepared saliva sample was analyzed using a glassy carbon electrode (GCE) modified with Sp-AuNPs.

## 3. Results and Discussion

### 3.1. Material Characterizations

#### 3.1.1. UV-Vis and X-ray Diffraction (XRD) Studies

**Figure 3:**
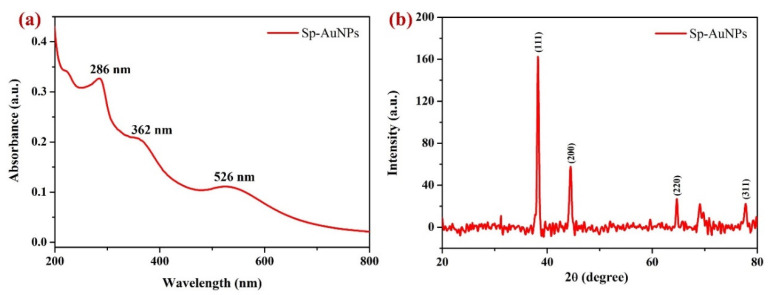
(**a**) UV-vis spectrum and (**b**) XRD pattern of synthesized Sp-AuNPs.

As shown in Figure 3a, the UV-vis spectrum of the Sp-AuNP dispersion was observed to have a localized surface plasmon resonance (LSPR) band at 526 nm. It is a distinct optical phenomenon observed in AuNPs, which involves an oscillating group of electrons in the conduction band (CB) of AuNPs, resonating at a specific wavelength of incident light [65,66]. The absorption spectrum of the synthesized Sp-AuNPs exhibited three distinctive peaks at 286 nm, 362 nm, and 526 nm (Figure 3a). The absorption peak at 526 nm was caused by the surface plasmon absorption of spherical AuNPs [67], indicating their presence. The approximate sizes of the AuNPs were determined from the absorption wavelength of AuNPs in the UV-Vis spectrum using the well-established LSPR concept as 25 ± 5 nm [68,69]. These results confirmed the formation of Au(0) nanoparticles. Furthermore, absorbance peaks found in the UV region at 286 nm and 362 nm indicated the π − π* transition of the fluorobenzene group and the n − π* as well as π − π* transitions of the quinolone ring of sparfloxacin, respectively [14,70].

Next, a non-destructive XRD method was utilized to investigate the crystallinity of the synthesized Sp-AuNPs. Figure 3b shows the XRD pattern of Sp-AuNPs, which exhibited four distinct peaks (2θ values) at 38.2°, 44.4°, 64.6°, and 77.7° that correlated with hkl values of (111), (200), (220), and (311), respectively. These observed peaks indicated that the produced Sp-AuNPs possess an FCC (face-centered cubic) lattice structure, which matched with JCPDS card no. 04-0784 [71,72]. The XRD peak at 38.2° was significantly greater in intensity compared to other peaks at 44.4°, 64.6°, and 77.7°. The prominent diffraction peak at 38.2° demonstrated that the reduced Au^0^ preferentially grew in the direction of (111). This refers to molecular-sized solids which were made up of a recurring 3D pattern of molecules or atoms with identical distances between each component. An XRD pattern like this is a typical of pure Au nanocrystals [73]. The average crystalline size of Sp-AuNPs was determined using the Debye–Scherrer equation (Equation (1)), considering the widths of the (111), (200), (220), and (311) Bragg reflections [74]. The average crystalline size of AuNPs was determined by XRD pattern as 23.6 nm (Table 1), which also matched with the results obtained by FE-SEM analysis.

(1) $$D=\frac{K\lambda }{\beta \cos\theta }$$

where *D* defines the crystalline size (nm) of the nanoparticles, *K* denotes the Scherrer constant or shape factor (0.98), λ is the X-ray wavelength (1.54 Å), β represents the full width at half maximum (FWHM, in radians), and θ denotes Bragg’s angle.

#### 3.1.2. Surface Morphology, Composition and Elemental Analysis

**Figure 4:**
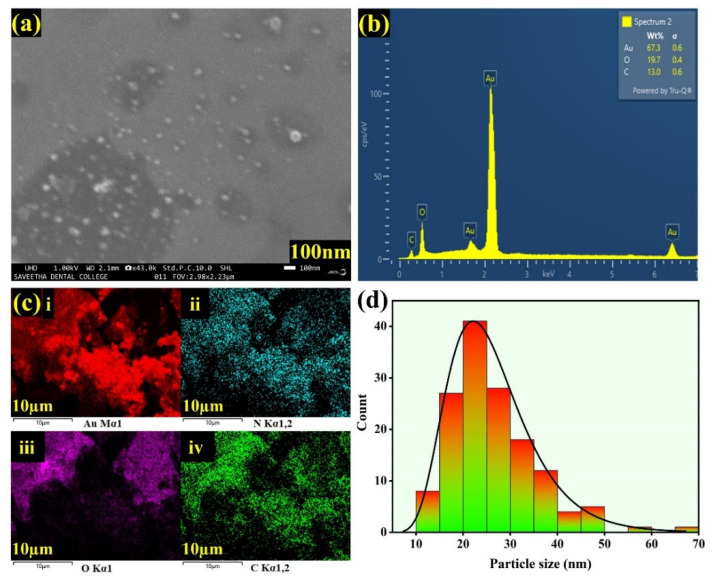
(**a**) FE-SEM image and (**b**) EDS spectrum of Sp-AuNPs. (**c**) Elemental mappings carried out on Sp-AuNPs: (i) gold, (ii) nitrogen, (iii) oxygen, and (iv) carbon. (**d**) Particle size distribution of Sp-AuNPs.

The structure, morphology and particle size of the synthesized Sp-AuNPs were investigated using FE-SEM. Figure 4a shows a FE-SEM image of Sp-AuNPs with spherical structures in the form of small particles. Our new synthesis method produced AuNPs with average sizes of 25 ± 5 nm. Additionally, EDS analysis confirmed the relevant elements in the Sp-AuNPs, with atomic percentages of Au (67.3%), C (13.0%), and O (19.7%), as shown in Figure 4b, providing further proof of the effective synthesis of Sp-AuNPs. Figure 4c shows the e-mapping images of the sample, which showed a homogeneous distribution of elements on the Sp-AuNPs. E-mapping analysis indicated an abundance of C, N, O, and Au, which correlated with sparfloxacin and AuNPs, respectively. This finding validated the utilization of sparfloxacin as a reducing agent in the synthesis process. The size distribution of spherical nanoparticles found in the FESEM image was analyzed using Image-J software. As shown in Figure 4d, the sizes of the majority of AuNPs were in the range of 20 to 30 nm.

#### 3.1.3. DLS and Zeta Potential Analysis

**Figure 5:**
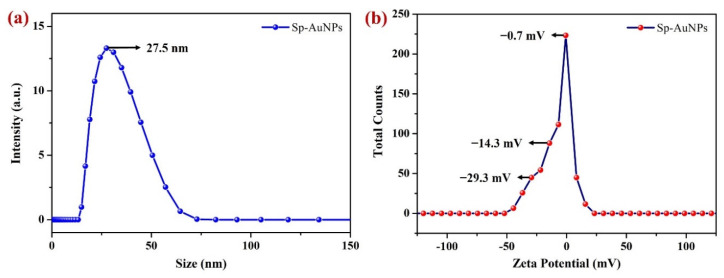
(**a**) DLS spectrum (particle sizes) and (**b**) Zeta potential of colloidal Sp-AuNPs.

The DLS technique was employed to determine the hydrodynamic diameter or size of the Au nanoparticles in a colloidal solution. Particle sizes acquired using DLS measurements are often bigger than those obtained with FE-SEM, as electron microscopy offers estimates of particle sizes in the dry condition [75]. DLS measurements of the Sp-AuNPs are depicted in Figure 5a, which reveals the average size of Sp-AuNPs as 27.5 nm. DLS observation showed a single peak, indicating that there was minimal agglomeration and a homogenous or monodisperse distribution of AuNPs in the solution. However, under specific storage conditions, such as pH, ionic strength, and temperature of the solution, agglomeration may occur [76,77].

The Zeta potential of homogenous dispersion of the Sp-AuNPs was measured as depicted in Figure 5b. This technique was utilized to investigate the surface charge and stability of Sp-AuNPs [78]. The data showed that the surface charge of the Sp-AuNPs particles ranged from −30 mV to −0.7 mV. The high intensity peak that appeared at −0.7 mV was most likely caused by Na^+^ ions accumulated on the Sp-AuNP surface (NaOH was used to maintain an alkaline pH of the solution during the synthesis of Sp-AuNPs). This result also indicated the existence of negative charges on the surface of Sp-AuNPs. The negative charge was mainly caused by the functional groups of sparfloxacin, which served as a stabilizing (by preventing their aggregation) and reducing agent.

### 3.2. Electrochemical Analysis of Sp-AuNPs

#### 3.2.1. Cyclic Voltammetry (CV) and EIS

**Figure 6:**
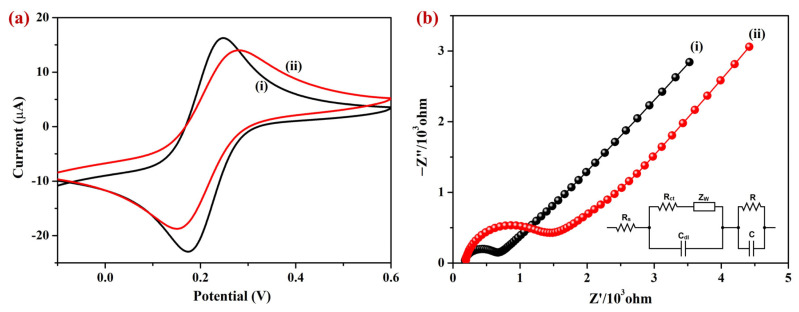
(**a**) CVs and (**b**) EIS spectrum of (i) A-SPE and (ii) Sp-AuNPs/A-SPE in 0.1 M KCl containing 2 mM [Fe(CN)_6_]^3−^. Inset: Randle’s equivalent circuit for the EIS spectra.

Using CV and EIS, the electrochemical characteristics of the Sp-AuNPs were studied in 0.1 M KCl containing 2 mM [Fe(CN)_6_]^3−^. Firstly, CV was performed using a A-SPE in 2 mM [Fe(CN)_6_]^3−^, obtaining cathodic (E_pc_) and anodic (E_pa_) peak potentials of 0.175 V and 0.247 V, respectively (Figure 6a, black curve). On the Sp-AuNPs/A-SPE, the E_pa_ and E_pc_ of [Fe(CN)_6_]^3−^ were 0.280 V and 0.154 V, respectively. When compared to the A-SPE, the Sp-AuNPs/A-SPE showed a lower peak current (about 15.05%) for the anodic (I_pa_) and cathodic (I_pc_) peaks of [Fe(CN)_6_]^3−^ (Figure 6a, red curve). This redox current decrease could be ascribed to electrostatic repulsion between Sp-AuNPs and [Fe(CN)_6_]^3−^ [79], indicating the existence of negative charges on the surface of the AuNPs (due to the presence of sparfloxacin on the surface of the AuNPs).

Nyquist plots were also recorded using the A-SPE (curve i) and Sp-AuNPs/A-SPE (curve ii) (Figure 6b) to evaluate the R_ct_ (interfacial electron transfer resistance) of the developed sensors [80]. The R_ct_ value of each electrode was determined from the Nyquist plots considering the diameter of the semicircle that appeared in the high-frequency zone. Among these, the Sp-AuNPs/A-SPE had a higher R_ct_ (1196 Ω) than the bare A-SPE (455.8 Ω). The larger semicircle suggested that the charge transfer resistance of Sp-AuNPs had increased, probably due to the electrostatic repulsion generated by the negatively charged sparfloxacin-capped surface of AuNPs [79]. Further, EIS spectra were processed using ZSimpWin 3.2 software, and the impedance values were matched with an appropriate equivalent circuit model, as shown in the inset of Figure 6b. The equivalent circuit comprised a combination of a CPE (constant phase element) and an RC (resistance/capacitance) circuit. Within this circuit, the symbols (W) and (C_dl_) were used to represent Warburg resistance and double layer capacitance, respectively. EIS results indicated that Sp-AuNPs possess electronegativity, which can be attributed to the existence of sparfloxacin on the AuNPs.

#### 3.2.2. Electrochemical Oxidation of Ethanol (EtOH)

**Figure 7:**
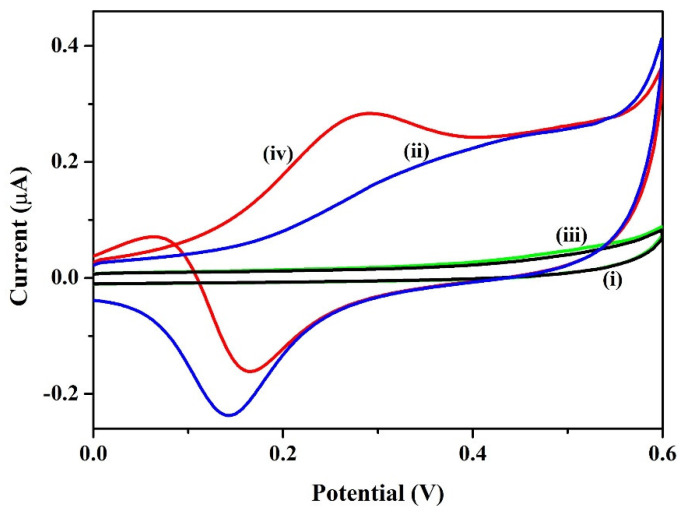
CVs of (i) A-SPE and (ii) Sp-AuNPs/A-SPE in 0.1 M NaOH, and the CVs curve of (iii) A-SPE and (iv) Sp-AuNPs/A-SPE in 0.1 M NaOH containing 17 mM EtOH at the scan rate of 20 mV s^−1^.

CVs were performed on the A-SPE (curves iii) and Sp-AuNPs/A-SPE (curve iv) in 0.1 M NaOH with 17 mM EtOH at a scan rate of 20 mV s^−1^ (Figure 7). In the absence of EtOH, the CV curve of the Sp-AuNPs/A-SPE showed a higher background current with a redox peak of AuNPs (the creation of Au oxides is attributed to these voltammetric peaks) between 0.1 V and 0.5 V (E_pa_ at 0.4 V and E_pc_ at 0.15 V) (Figure 7 curve ii). As expected, the A-SPE did not show any redox peak and the background current was also lower compared to the Sp-AuNPs/A-SPE (Figure 7 curve i). However, when EtOH (17 mM) was added, the Sp-AuNPs/A-SPE produced an oxidation peak of EtOH at 0.28 V (E_pa_) with a higher peak current of 0.29 µA, and the reduction peak current of the AuNPs was decreased (−0.17 µA), while the reduction peak potential shifted slightly towards the positive side at 0.16 V (E_pc_), indicating that gold oxides (electrogenerated) were involved in the electrocatalytic oxidation process of EtOH. The observed catalytic effect was mainly due to surface oxides, which were produced during the oxidation process of the preadsorbed monolayer [81]. Moreover, there was no redox peak observed in the A-SPE, and the background current of the A-SPE remained the same (curve iii) before and after the addition of 17 mM EtOH, which indicated that the A-SPE may not be useful for EtOH oxidation without AuNPs.

**Figure 8:**
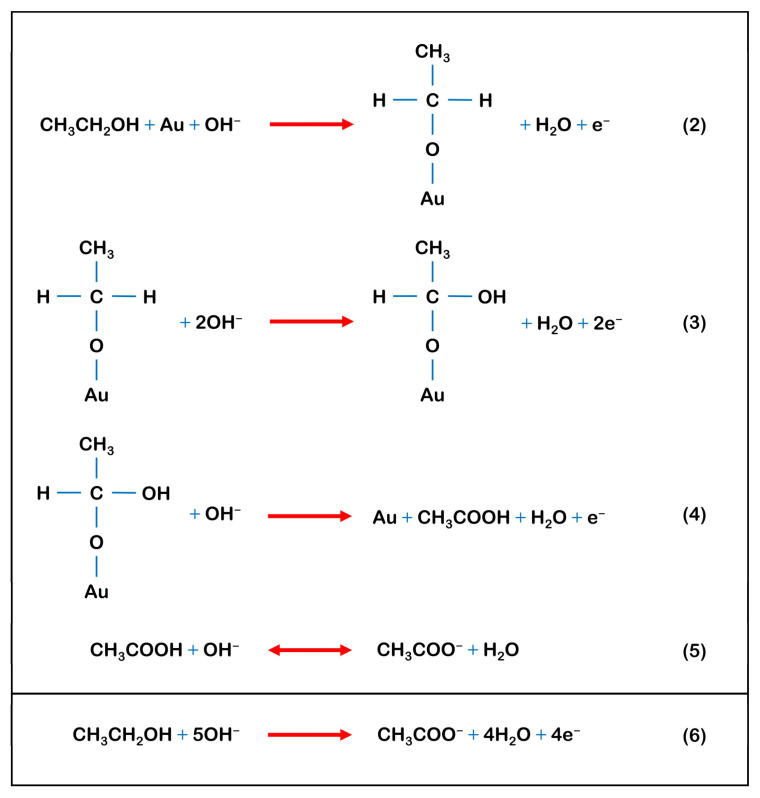
Schematic representation of ethanol oxidation processes on Sp-AuNPs/A-SPE in alkaline media.

Figure 8 depicts the electro-oxidation mechanism of EtOH on the Sp-AuNPs/A-SPE [82]. In this schematic representation, the initial adsorption (Equation (2)) happens through an ethoxy intermediate; the alcohol molecule releases one electron upon interacting with hydroxyl ions to form a water molecule and ethoxy. Subsequently, the ethoxy molecule adsorbs onto the Au surface. Unlike the original form, the interaction between the adsorbed ethoxide and non-adsorbed OH species occurs through the Eley–Rideal mechanism (Equation (3)) [83]. In this scenario, an activated complex form near the electrode surface between the adsorbed and other non-adsorbed species. This mechanism suggests that as the concentration of one of the two reacting species (either adsorbed or non-adsorbed) increases, the reaction rate rises to a specific limit, with no decrease expected at higher concentrations. The C-H bond on the alpha-carbon rifts during the initial adsorption stage of the primary alcohols in alkaline environment (Equation (4)). Furthermore, the interaction between the alcohol molecule and the hydroxyl ions at the interface results in the formation of a symmetric surface transition state [84,85,86]. Equations (5) and (6) describe a reversible reaction involving acetic acid or acetate ions with the overall electro-oxidation reaction of ethanol, which was based on the mechanism proposed by Termiliosi-Filho et al., who had confirmed it by comprehensive chromatographic analysis with a polycrystalline gold electrode in alkaline media. Their work confirmed that around 4.25 electrons were transferred during this process. Furthermore, acetic acid (or acetate ions in alkaline media) was identified as the major product [87].

#### 3.2.3. Determination of EtOH

**Figure 9:**
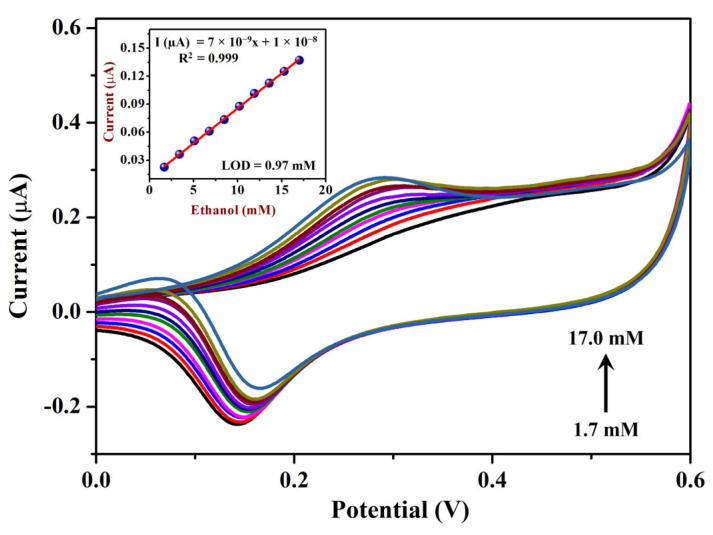
CVs performed with various concentrations of EtOH from 1.7 mM to 17 mM using Sp-AuNPs/A-SPE in a 0.1 M NaOH at a scan rate of 20 mV s^−1^. Inset: calibration graph of EtOH, illustrating the relationship between different EtOH concentrations (mM) and the corresponding I_pa_ of EtOH (µA).

**Figure 10:**
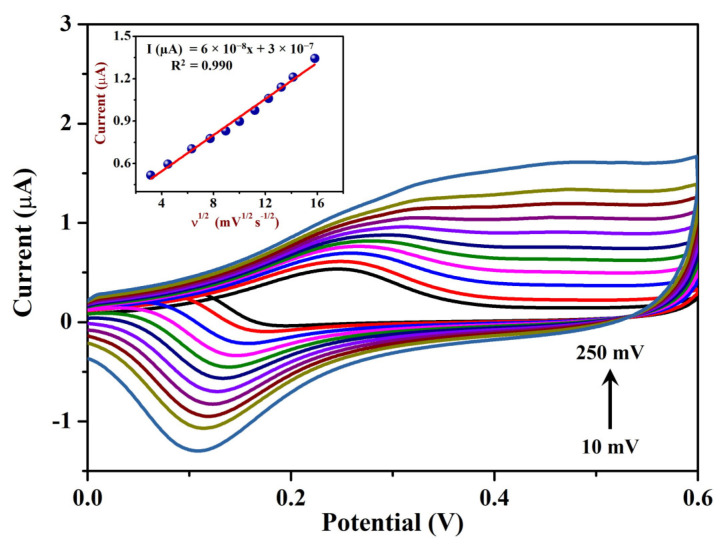
Sp-AuNPs/A-SPE used to record CVs at different scan rates in 0.1 M NaOH containing 17 mM of EtOH. Inset: relationship between the square root of scan rate (mV s^−1^) and the I_pa_ of EtOH.

The contribution of the surface oxides of AuNPs in the catalytic oxidation of ethanol was demonstrated by measuring the voltammetric response to different EtOH concentrations [88]. CVs were recorded in 0.1 M NaOH with various EtOH concentrations (from 1.7 to 17 mM) using the Sp-AuNPs/A-SPE at a scan rate of 20 mV s^−1^. Figure 9 shows that the oxidation peak currents (I_pa_) increased linearly with the concentrations of EtOH (at +0.28 V). A linear regression plot was made to correlate the concentration of EtOH with the corresponding response current on the Sp-AuNPs/A-SPE (inset of Figure 9). The I_pa_ for each concentration of EtOH was measured at +0.28 V after subtracting the blank current. As the ratio of EtOH in the solution increases, the cathodic peak associated with surface Au oxide reduction decreases gradually, confirming the role of surface oxides in the catalytic process [88]. This characteristic can be explained by the increased availability of EtOH molecules which interacted with the gold oxides through the early phases of electrochemical reduction [82]. These findings indicate that the Sp-AuNPs/A-SPE exhibited high sensitivity and stability during the electro-oxidation of EtOH. Additionally, to investigate the impact of scan rates (ν) on EtOH oxidation, CVs were carried out on the Sp-AuNPs/A-SPE in 0.1 M NaOH containing 17 mM EtOH at different scan rates from 10 to 250 mV s^−1^ (Figure 10). The oxidation peak current (I_pa_) of EtOH exhibited a linear relationship with ν^1/2^. As ν increased, the I_pa_/I_pc_ ratio decreased, indicating that the catalytic process was associated with a surface-confined mediator (specifically surface oxide) [88,89]. The reduction wave diminished with lower scan speeds, whereas a minor anodic wave was visible during the reverse scan. The oxidation peak potential (E_pa_) of EtOH gradually shifted towards the positive side, which was indicative of a kinetic-controlled and irreversible electrochemical reaction [90,91]. The linear equation obtained from the data was Y = 6 × 10^−8^x + 3 × 10^−7^, with the correlation coefficient of (R^2^) of 0.990 (inset of Figure 10).

#### 3.2.4. Differential Pulse Voltammetry (DPV) Studies

**Figure 11:**
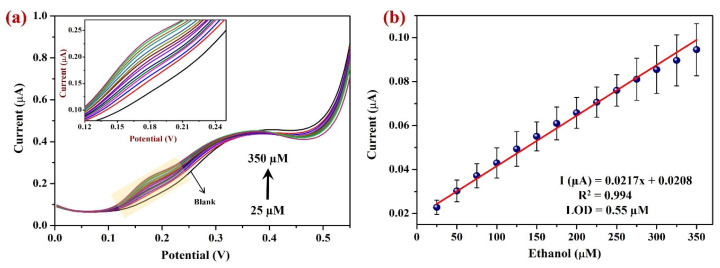
(**a**) DPV measurements were performed using Sp-AuNPs/A-SPE in 0.1 M NaOH, with various EtOH concentrations ranging from 25 µM to 350 µM. The highlighted portion of (**a**) has been expanded and indicated for clarity as inset. (**b**) A linear calibration curve was plotted between the oxidation peak current response vs. various EtOH concentrations. Error bars indicate the average value with standard deviation of three measurements (n = 3).

DPV was employed to study the linear determination of EtOH on the Sp-AuNPs/A-SPE in the potential range of 0 to 0.55 V in a 0.1 M NaOH [92,93,94]. The Sp-AuNPs/A-SPE exhibited a linear current response when the EtOH concentrations were increased from 25 µM to 350 µM (Figure 11a), with a sensitivity of 0.31 µA µM^−1^ cm^2^. In dynamic linear range measurements, DPV demonstrated higher precision than CV [95,96]. The observed high sensitivity was due to the rapid electrocatalytic oxidation of EtOH aided by the Sp-AuNPs/A-SPE. The calibration curve (Figure 11b) was plotted between the I_pa_ of EtOH and various concentrations of EtOH, where the linear equation was determined as Y = 0.021x + 0.0208 with an R^2^ value of 0.994. The LOD (limit of detection) was calculated using the formula of LOD = 3.3 × σ/s, where σ represents the standard deviation (3.67 × 10^−9^ A µM^−1^) and s represents the slope of the calibration curve (0.0217 × 10^−6^ A). The resulting LOD was determined to be 0.55 µM. It should be noted that at higher EtOH concentrations (over 350 µM), the steady-state current begins to drop due to possible saturation of the modified electrode’s active catalytic regions. The proposed method’s performance was also compared with other existing EtOH sensors in Table 2, which displays the sensitivity, linear range, and LOD of the EtOH. It was clear that the performance of our proposed EtOH sensor was comparable to that of other reported sensors.

#### 3.2.5. Repeatability, Reproducibility, and Stability Studies

**Figure 12:**
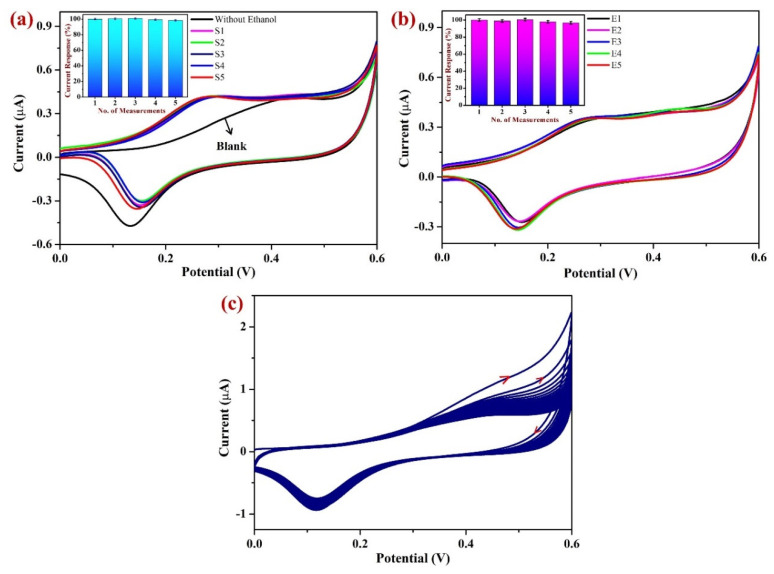
(**a**) Repeatability measurements of 20.4 mM EtOH using the same Sp-AuNPs/A-SPE for up to five repeated measurements in 0.1 M NaOH at a scan rate of 20 mV s^−1^. Inset shows the bar diagram of the I_pa_ response of EtOH for each measurement vs. no. of measurements. (**b**) Reproducibility measurements of 20.4 mM EtOH using five independent Sp-AuNP-modified A-SPEs in 0.1 M NaOH at a scan rate of 20 mV s^−1^. Inset shows the bar graph of the I_pa_ response of EtOH at each electrode vs. no. of measurements. (**c**) Stability analysis of the Sp-AuNP-modified A-SPEs in 0.1 M NaOH was carried out using CV after 50 consecutive cycles at a scan rate of 50 mV s^−1^. Error bars indicate the mean value with a standard deviation of five repeated measurements (n = 5) (**a**) and five individual measurements (n = 5) (**b**).

In order to determine the sensor’s accuracy and the repeatability of the modified electrode, the freshly prepared Sp-AuNPs/A-SPE was used to investigate the oxidation of 20.4 mM EtOH in 0.1 M NaOH (Figure 12a). The inset of Figure 12a clearly shows the differences in the I_pa_ of EtOH at +0.28 V. During the first three independent observations, the I_pa_ of EtOH showed a minor increase at +0.28 V.

Following the fourth and fifth consecutive measurements, the I_pa_ of EtOH began to fall, possibly as a consequence of material leaching from the A-SPE after the third measurement (before each measurement, the modified electrode was gently rinsed in DI water). These findings indicate that the Sp-AuNPs/A-SPE was more stable after repeated measurements.

Moreover, in order to assess the reproducibility of the sensor, five individual Sp-AuNP-modified A-SPEs were prepared and utilized to determine 20.4 mM EtOH in 0.1 M NaOH (Figure 12b). The inset of Figure 12b clearly displays the variations in the oxidation peak current (I_pa_) of EtOH at +0.28 V. The I_pa_ of EtOH showed slight variations at +0.28 V after measurement with the five individual modified electrodes. The average current response of the five modified electrodes was found to be 98.7%. These results showed that the Sp-AuNP-modified A-SPE exhibits highly reproducible properties. Similarly, the sensor’s stability was evaluated using CV on a newly prepared Sp-AuNPs/A-SPE. The CV was also recorded with 50 consecutive cycles using the Sp-AuNPs/A-SPE in 0.1 M NaOH at a scan rate of 50 mV s^−1^. As depicted in Figure 12c, the modified electrode exhibited enhanced stability with small variation in peak currents after the 10th cycle. This finding indicates that colloidal Sp-AuNPs are relatively stable and can be utilized to develop robust sensors for EtOH detection.

#### 3.2.6. Interference and Real Sample Analysis

**Figure 13:**
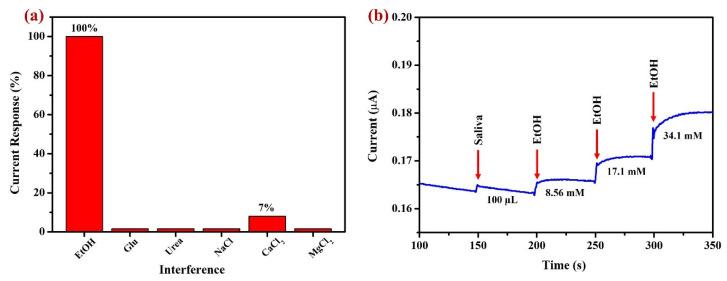
(**a**) The bar plot shows the current response changes after the addition of interfering substances to a solution containing 8.56 mM EtOH. The Sp-AuNPs/GCE was used for this interference study at 0.32 V in the presence of 8.56 mM EtOH in 0.1 M NaOH. (**b**) To calculate the recovery percentage, an amperometry (i-t) curve was obtained using a Sp-AuNPs/GCE with a saliva sample and various concentrations of standard EtOH solutions in 0.1 M NaOH. During these measurements, 0.1 M NaOH was stirred at 750 rpm.

Amperometry (AMP, i-t curve) was conducted for interference analysis to assess the selectivity of Sp-AuNPs in ethanol detection (Figure 13a). Sp-AuNPs were modified on a glassy carbon electrode (GCE with diameter of 0.07 cm^2^). This experiment was carried out with the addition of potential interfering substances to a solution containing 8.56 mM ethanol in 0.1 M NaOH at an applied potential of +0.32 V with constant stirring at 750 RPM to check whether these substances interfere with the ethanol detection. In this study, glucose (Glu), urea, sodium chloride (NaCl), calcium chloride (CaCl_2_), and magnesium chloride (MgCl_2_) were added, and they did not show any interference, while ascorbic acid (AsA) and uric acid (UA) exhibited significant interference. All these interference solutions were used at a 0.5 mM concentration except for AsA (5 µM) and UA (5 µM). These compounds were selected for selectivity assessment due to their common presence in human saliva samples [114,115,116]. These results indicated that most inorganic and some organic compounds (Glu and urea) did not interfere, but AsA and UA interfered likely due to the non-specific adsorption on AuNPs [117,118]. To mitigate these interferences, various strategies including surface modification and incorporation of other nanomaterials to form composites may be helpful in developing highly selective ethanol sensors in the future. Additionally, the Sp-AuNPs/A-SPE sensor may be well suited for the sensitive detection of ethanol in non-organic samples, allowing for the estimation of alcohol concentration in liquors to verify their purity.

The real sample and recovery analyses were conducted by AMP (i-t curve) using a Sp-AuNPs/GCE at +0.30 V. Three independent measurements (n = 3) were made for each sample (Table 3). These experiments were carried out on a diluted saliva sample, as illustrated in Figure 13b. Initially, 100 µL of saliva was mixed with 0.1 M NaOH, then standard (std) EtOH was spiked into the mixture at three different concentrations of 8.56 mM, 17.1 mM, and 34.1 mM. To determine the unknown EtOH concentration in the saliva sample, AMP recorded the std EtOH (without saliva sample) using the Sp-AuNPs/GCE at +0.30 V in 0.1 M NaOH. The std EtOH measurement was then used to calculate the unknown concentrations of ethanol present in the saliva. The results showed that the Sp-AuNP-modified electrode exhibited a current response upon the addition of the saliva sample, possibly due to the presence of some of the interfering molecules. After the addition of std EtOH, the current responses were recorded. Table 3 summarizes the oxidation current response of the saliva sample before and after spiking it with EtOH, which allowed us to calculate the recovery of the spiked EtOH in the saliva sample (99.6%–103.1%). These results concluded that the detection of EtOH in 0.1 M NaOH can be carried out using Sp-AuNPs in saliva.

## 4. Conclusions

An electrochemical sensor based on Sp-AuNPs was prepared for the first time to accurately detect ethanol in 0.1 M NaOH and exhibited excellent sensitivity and stability. FE-SEM, E-mapping, EDS, UV-visible, and XRD analyses of the synthesized Sp-AuNPs revealed the successful formation of homogenous AuNPs with a well-distributed elemental composition. DLS and Zeta potential tests also revealed that the synthesized Sp-AuNPs had an average particle size of 25 ± 5 nm and a negative surface charge, which was attributed to the existence of sparfloxacin on AuNPs surface. A Sp-AuNPs/A-SPE-based electrochemical sensor was then developed for EtOH detection and exhibited higher oxidation peak currents than a bare SPE. Thus, the newly developed Sp-AuNPs demonstrated their potential for effective ethanol determination in various samples. The Sp-AuNPs/A-SPE showed a linear response for EtOH determination from 25 to 350 µM, with the lowest LOD of 0.55 µM. Furthermore, this sensor demonstrated its capability to sense ethanol under alkaline condition, outperforming previous studies (see Table 2). The results of reproducibility and repeatability studies had revealed that Sp-AuNPs/A-SPE was highly stable and very sensitive for in situ EtOH detection. Additionally, the electrochemical detection of EtOH was conducted in the presence of interfering substances in alkaline media. Furthermore, human saliva samples were tested and the recovery rate of spiked EtOH in these samples was determined to be 99.6%. Finally, the Sp-AuNPs-based sensor exhibited excellent sensitivity to the precise and direct determination of EtOH concentration. However, further surface modification is required to attain higher selectivity for ethanol detection in other biological or food samples.

## Figures and Tables

**Table 1:** Average crystalline size of AuNPs calculated using Debye–Scherrer equation.

Hkl Value	2θ	FWHM	β = π/180 × FWHM	Cos θ	Crystalline Size–D (nm)
111	38.23	0.3919	0.0068399	0.9448	23.3
200	44.42	0.5082	0.0088697	0.9258	18.3
220	64.67	0.3576	0.0062412	0.8449	28.6
311	77.75	0.4562	0.0079621	0.7785	24.3
		Average crystalline size (nm)			23.6

**Table 2:** Comparison of analytical data obtained from various electrochemical sensors reported for EtOH along with the current method (Sp-AuNPs/A-SPE).

Sensing Material	Method	Sensitivity (μA mM^−1^ cm^−2^)	Linear Range (mM)	LOD (µM)	References
ZnO-CeO_2_	I-V characteristics	0.833	0.17–17.04	160	[97]
Mesoporous Pd–ZnO	I-V characteristics	33.08	0.05–0.8	19.2	[98]
Gd_2_O_3_	I-V characteristics	0.266	0.17–850	52.2	[99]
Mg (OH)_2_ hexagonal nano disks	I-V characteristics	6.89	0.0001–10	0.073	[100]
CuO nanosheets	I-V characteristics	0.972	0.17–1700	143	[101]
Mg (OH)_2_ nanosheet	I-V characteristics	3.99	0.01–1000	5	[102]
Mesoporous Ag/α–Fe_2_O_3_	I-V characteristics	2.93	0.8–15	15.4	[103]
SnO_2_ doped ZnO	I-V characteristics	62.56	0.195–25	137	[104]
Gr/In_2_O_3_	CV	–	100–1200	68 mM	[105]
ADH/Gr/GCE	AMP	–	0.2–21	25	[106]
rGO/In-en	CV	–	0.1–3000	100	[107]
Au-AgNPs/P(L-Cys)-rGO	C-AMP	0.644	0.017–658	5	[108]
IL-graphene/chitosan/ADH	C-AMP	6.91	0.025–0.2	5	[109]
NP–Pt/Co alloy	AMP	-	0.2–12	8	[110]
NP–Pt/Ni	AMP	-	0.2–11	10	[111]
PGE/SWCNTs/PCV/ADH	AMP	1.94	0.009–0.32	-	[112]
Pd–Ni/Si-NWs	AMP	0.76 mA	0–20.4	10	[113]
Sp-AuNPs	DPV	0.31 µA µM^−1^ cm^2^	0.025–0.35	0.55	Current work

Footnotes: ZnO—zinc oxide, CeO_2_—cerium dioxide, Pd—palladium, Gd_2_O_3_—gadolinium oxide, Mg (OH)_2_—magnesium hydroxide, CuO—copper oxide, Ag—silver, α–Fe_2_O_3_—hematite phase of iron oxides, SnO_2_—tin oxide, Gr—graphene, In_2_O_3_—indium oxide, ADH—alcohol dehydrogenase, GCE—glassy carbon electrode, rGO—reduced graphene oxide, In-en—indium ethylenediamine, Au-AgNPs—gold–silver bimetallic nanoparticles, P(L-Cys)–Poly(L-Cysteine), IL-Gr—ionic liquid-functionalized graphene, NP-Pt—nanoporous platinum, Co—cobalt, Ni—nickel, PGE—pencil graphite electrode, SWCNTs—single-wall carbon nanotubes, PCV—pyrocatechol violet, Si-NWs—silicon nanowires, CV—cyclic voltammetry, AMP—amperometry, C-AMP—chronoamperometry, DPV—differential pulse voltammetry.

**Table 3:** Measurements of spiked EtOH concentrations in saliva samples with a Sp-AuNP-modified electrode.

S. No.	Samples	Added (mM)	Found (mM)	Recovery (%)	SD	RSD%
1	Human saliva	-	1.56	-	0.421	26.956
2	Spiked EtOH	8.56	10.09	99.68	0.456	5.347
3	Spiked EtOH	17.1	17.08	99.87	0.291	1.703
4	Spiked EtOH	34.1	35.19	103.18	0.324	0.922

## Data Availability

Data can be made available upon a valid request.

## Supplementary Materials

The following supporting information can be downloaded at: https://www.mdpi.com/article/10.3390/s23198201/s1, Figure S1: Cyclic voltammetry (CV) results of (i) bare SPE and (ii) A-SPE (activated SPE) in 0.1 M KCl containing 2 mM [Fe(CN)_6_]^3−^ at scan rate of 50 mV s^−1^; Figure S2: EIS spectra of (i) bare SPE and (ii) A-SPE (activated SPE) in 0.1 M KCl containing 2 mM [Fe (CN)_6_]^3−^ at an amplitude of 5 mV.
