# Supplementary material for: Using Sparfloxacin-Capped Gold Nanoparticles to Modify a Screen-Printed Carbon Electrode Sensor for Ethanol Determination
Source: Sensors (Basel). 2023 Sep 30;23(19):8201. doi: 10.3390/s23198201 (PMC10575339; doi:10.3390/s23198201)
Supplement: Supplementary file 1 [file sensors-23-08201-s001.zip › sensors-2550093-supplementary.pdf]

sensors

Supplementary Details

Using Sparfloxacin-Capped Gold Nanoparticles to Modify a Screen-Printed Carbon Electrode Sensor for Ethanol Determination

Vasanth Magesh [1,†], Vishaka S. Kothari [1,†], Dhanraj Ganapathy [1], Raji Atchudan [2,†], Sandeep Arya [3], Deepak Nallaswamy [1] and Ashok K. Sundramoorthy [1,*]

[1] Centre for Nano-Biosensors, Department of Prosthodontics, Saveetha Institute of Medical and Technical Sciences, Saveetha Dental College and Hospitals, 162 Poonamallee High Road, Velappanchavadi, Chennai 600077, India
[2] School of Chemical Engineering, Yeungnam University, Gyeongsan 38541, Republic of Korea
[3] Department of Physics, University of Jammu, Jammu 180006, India
* Correspondence: ashok.sundramoorthy@gmail.com
† These authors contributed equally.

Supporting Figures:

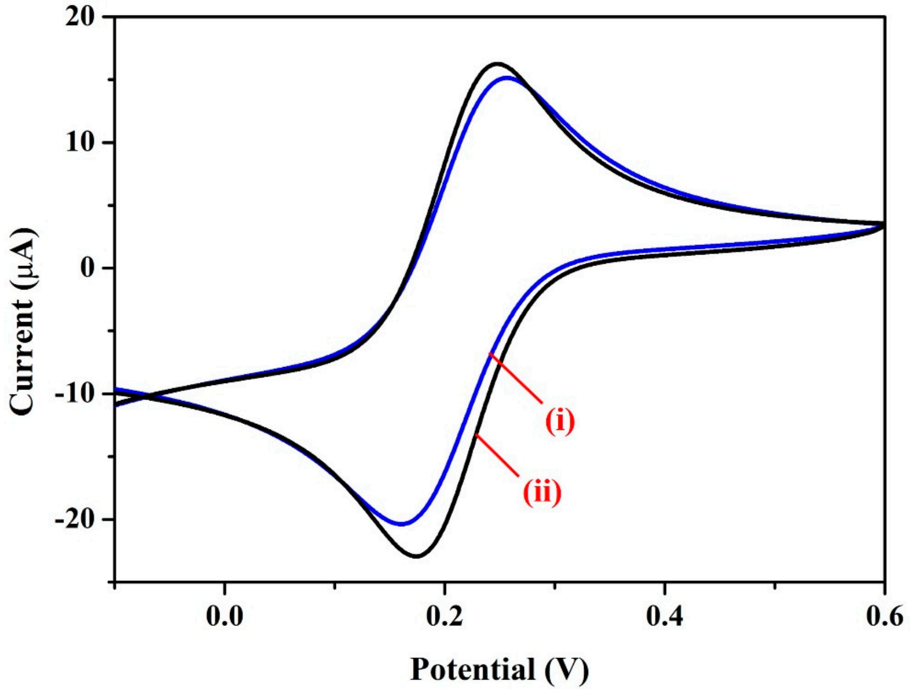

Figure S1. Cyclic voltammograms (CV) were recorded using (i) bare SPE and (ii) A-SPE (activated SPE) in 0.1 M KCl containing 2 mM $[Fe(CN)_6]^{3-}$ at a scan rate of 50 mV s^{-1}.

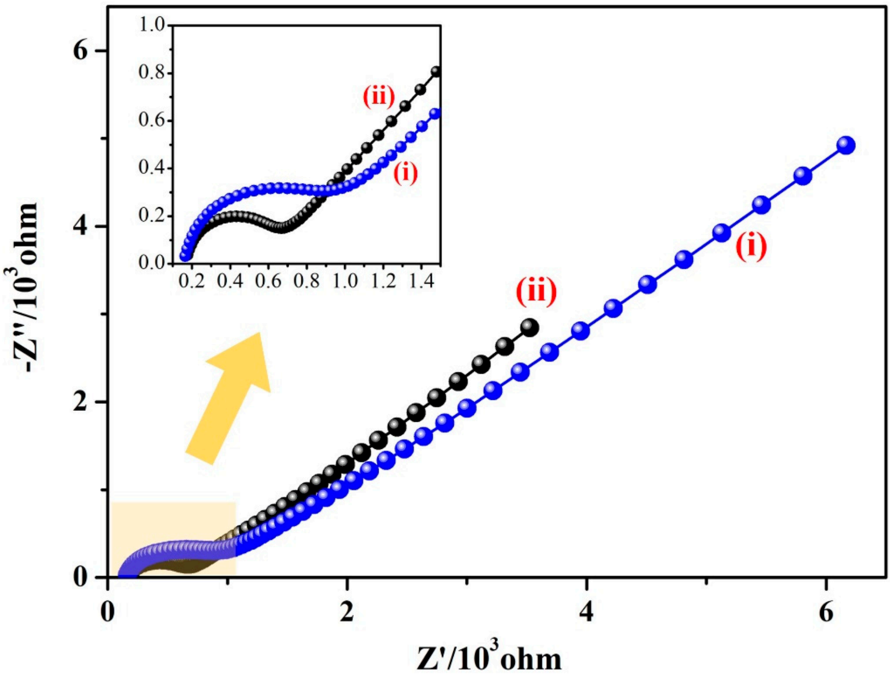

Figure S2. EIS spectrum of (i) bare SPE and (ii) A-SPE in 0.1 M KCl containing 2 mM $[Fe(CN)_6]^{3-}$ at an amplitude of 5 mV. Inset: Enlarged image of highlighted portion in Figure S2.
